# Rapid behavioral changes during early development in Peters’ tent-making bat (*Uroderma bilobatum*)

**DOI:** 10.1371/journal.pone.0205351

**Published:** 2018-10-24

**Authors:** Jenna E. Kohles, Rachel A. Page, Dina K. N. Dechmann, M. Teague O’Mara

**Affiliations:** 1 Smithsonian Tropical Research Institute, Balboa, Ancón, Panama City, Panama; 2 Department of Migration and Immuno-Ecology, Max Planck Institute for Ornithology, Radolfzell, Germany; 3 Department of Biology, University of Konstanz, Konstanz, Germany; 4 Zukunftskolleg, University of Konstanz, Konstanz, Germany; University of Western Ontario, CANADA

## Abstract

Bats transition from flightless, milk-sustained infants to volant, foraging juveniles in the span of a few weeks to a few months. This rapid development is accompanied by fast growth and weight gain, but behavioral development remains poorly understood. We addressed development of maternal support and pup independence for Peters’ tent-making bat (*Uroderma bilobatum*) in light of population level reproductive patterns. *Uroderma bilobatum* exhibited seasonal bimodal polyoestry at our study site. Births occurred over one month within a reproductive bout, resulting in variable levels of behavioral development for pups in the same maternity group. Pups reached adult forearm length more quickly than adult mass, facilitating the ontogeny of flight. Maternal support consisted of nursing and thermoregulation, transporting pups between night and day roosts, and milk provisioning between foraging bouts. We did not observe provisioning with solid food. Pups interacted only with their own mother. Between 25 to 40 days into a reproductive bout they matured by suckling progressively less and fledging over multiple nights in a two-stage process assisted by mothers. We describe several parturition events as well as a novel form of stereotyped tactile stimulation involving forearm pulses by mothers against suckling pups that may serve to promote weaning. Rapid behavioral changes in both pups and mothers accompany pup morphological development through maturation.

## Introduction

Early development is a vulnerable and demanding life phase. Both mammal and bird species depend on parental care for much of this period, but mammals must wean from mothers’ milk while birds must fledge from the nest in the transition to adulthood. Bats experience both developmental challenges. They must shift from feeding only on mother’s milk to finding and consuming solid food in conjunction with their first sustained flights. Developmental changes that accompany this drastic transition are not well understood.

The early developmental period of bats is distinctive because bats deviate from many patterns of small mammal life history [1]. Small mammals typically mature early, produce only a few large litters, and experience high natural mortality [2]. In contrast, most bat species invest heavily in one offspring at a time, raising pups to near adult size, and they have many reproductive events over a relatively long lifetime [3–5]. Most of these traits are attributed to adaptations for flight [5,6]. A mother’s wing load increases with litter mass and with increasing mass of the neonate, constraining the number and size of pups she can carry in utero while maintaining the ability to fly and effectively forage [7].

Early developmental traits vary widely across bat species. Pup mass at birth ranges from 17.5 to 28.8 percent of mother’s mass and mothers suckle offspring between 21 and 300 days [5,6,8]. Their forearms, as standard size measure, lengthen at rates of 0.4–2.0 mm day^-1^ and they accumulate mass at 0.1–1.3 g day^-1^ [9]. Some insectivorous species become volant at just two weeks of age while large frugivores may take up to twelve weeks [9]. In most species, bat pups cannot fledge until they reach 90 percent of adult forearm length and thus grow rapidly to achieve flight and foraging independence, relieving mothers of nutritional support [10]. Behavioral changes accompanying physical development have rarely been described, especially during fledging and weaning [11–14]. Mother-offspring conflict in particular is thought to be uncommon in bats, but is rarely addressed by bat biologists [6].

We investigated the breeding biology and early developmental period for one of the most common leaf-nosed bats in Central America, Peters’ tent-making bat (*Uroderma bilobatum)*. *Uroderma bilobatum* modify leaf structures to form roosting tents [15]. They roost in semi-stable groups ranging from 2 to 50 individuals and are specialized on fruiting figs [16]. Roosts serve as information centers, where individuals can learn about available food sources from roostmates [17,18]. Females form maternity groups that exhibit relatively synchronous parturition [19]. We aimed to address behavioral means of maternal support and pup independence and how these change during early development for *U*. *bilobatum*. We combined population level reproductive patterns with behavioral observations at the roost, to place behavior in the context of the birthing season and pup morphological development. Behavioral changes accompanying early development may elucidate ontogeny of flight, foraging, and reproductive strategies for *U*. *bilobatum* and related species.

## Methods

### Birthing season

At our study site, the village of Gamboa, Panama (N 09.07; W 079.41), *U*. *bilobatum* roost under the eaves of overhanging roofs of houses [18]. Observers visited 30 houses during the day once per week from June 2013 through July 2014 (gap in October 2013) and counted the number of adults and pups using binoculars. An additional 37 houses were surveyed opportunistically. In our survey, a house designates one roost. Many roosts contained multiple small clustered groups of bats roosting in separate roof partitions. Pups were distinguished from adults by their smaller size and darker color.

### Morphological development in pups

Bats were captured on 11 dates from 02 August 2012 to 21 August 2013. We used hand nets to capture roosting groups under the eaves of houses and mist nets to capture foraging bats in the forest. We recorded mass to the nearest 0.1 g, forearm length to the nearest 0.1 mm, sex, and roosting location, and tagged individuals with a subcutaneous passive integrated transponder (ID 100, Trovan Inc.). We estimated age of pups as the number of days from the start of the birthing period at their roost to their capture date. The start of the birthing period was determined from the roost survey as the date infants were first seen at the roost for the given reproductive bout. For juveniles captured outside of roosts or at roosts for which survey data was incomplete, we used the date infants were first seen at any roost in the whole survey for the given reproductive bout. Births were not tightly synchronized between or within all roosts, so pup age may be overestimated by 15–30 days. We used captures of foraging adult *U*. *bilobatum* to calculate average adult mass and forearm length for each sex to compare with pup measurements.

### Behavioral development

We made behavioral observations at one maternity roost for a 2-week period spanning peak pup presence, from 6 July 2014 to 21 July 2014. A stable maternity group of 11–13 females and their pups, occupying a 1x1 m section of roof, was video recorded for 24-hour intervals using a Bell Howell DNV16HDZ Camcorder and a CMVision 48 LED IR Illuminator. The camera was initially set up at night while bats were absent from the roost and memory cards were replaced as infrequently as possible to minimize disturbance to the roosting group. Bats were not externally marked so individuals could not be distinguished in our recordings, although mother-pup pairs could be followed as a known unit for the length of each day roosting period. We assumed that pups suckled only from their genetic mother because mothers were never seen with two different pups on the same day.

Videos were scored by a single observer (JK) using SolomonCoder (András Péter, http://solomoncoder.com) in a frame-by-frame analysis at five frames per second. The videos were scored using an instantaneous scan sampling method adapted from Winchell and Kunz [20] and Altmann [21]. The observer scored instantaneous behavior for all pups every 10 s for 100 s (10 scans per session) separated by 10 min intervals (arrival and departure) or 15 min intervals (daytime). Subsets of the videos from all time periods were used to establish an ethogram (Table 1). Behaviors were divided into five categories: 1) position, 2) activity, 3) suckling, 4) social interactions [with roostmates besides mother], and 5) mother-pup interactions (Table 1). Observations were divided into three periods: 1) morning arrival (first bat arrival after 0500 H until 0730 H), 2) daytime (0730–1830 H), and 3) evening departure (1830 H until all bats left the roost). The time windows for arrival and departure were determined from preliminary video analysis as the periods in which all bats generally arrived or departed. Analyses also showed that once bats left the roost they did not return until the following morning arrival period. Thus, the period of night between departure and arrival was not scored.

**Table 1:** Ethogram.

Category	Behavioral Unit	Description
Position	With mother	*Pup is suckling*, *clinging to mother’s chest*, *or clustered tightly with her*
	Apart from mother	*Pup is not suckling and the majority of its body is not touching its mother*
	Mother absent	*Pup is not suckling; mother has moved away from pup in the roost or left the roost entirely*
Activity	Grooming	*Scratching body and/or lowering foot to mouth or licking wings and other parts of body*
	Shifting	*General movement in the roost including stretching*, *crawling*, *switching teats while suckling*
	Flapping	*Rapidly flapping wings while holding them outstretched*
	Resting	*Staying still in one position with wings tucked into body even while suckling*
Suckling	Suckling	*Mouth to mother’s chest*, *assumed to be suckling and attached to teat*
	Not Suckling	*Nose/mouth visibly unattached to mother’s teats; can still be clinging to mother’s body*
Social interactions [with roostmates besides mother]	Displace	*Pup’s interaction with another individual causes that individual to leave the roost*
	Nip	*Definitive agonistic contact with another individual via mouth*
	Head Incline	*Definitive lean/stretch lead with head in the direction of another individual with no contact*
	Nose-to-nose	*Neutral contact involving intentional nose to nose touching between two individuals*
	Nose-to-back	*Neutral contact involving intentional nose to back touching from one individual to the other*
	Disturb	*Any interaction towards another individual that causes a halt to that individual’s previous action/state*, *but does not fit into a more specific category*
	Not Social	*Not interacting with any other individuals*
	Other	*Any behavior involving other individuals that does not fall into another category*
Mother-pup interactions	Mother Grooming	*Mother licking or scratching any part of pup’s body and wings*
	Forearm Pulses	*Mother pulsing arms in a stereotyped forward and backwards motion against a pup that is clinging to her chest and suckling*

In addition to scan sampling in the arrival and departure periods, we noted the number of mothers carrying their pups and number of pups flying on their own for post-foraging arrival and pre-foraging departure. We calculated the latency to depart (the duration from the time of first bat departure from the roost) for each mother, mother-pup pair, or pup. A novel behavior was observed during departure in which mothers pulsed their forearms against pups before flying away from the roost (“forearm pulses”, Table 1, S1 Video). We identified all forearm pulsing events in the videos and used the focal sampling method to score all behaviors of both mothers and pups during the interaction period (time from first forearm pulse until last pup detachment or mother departure) [21].

Four births occurred during video recordings, one at the focal roost and three at a second roost, which was not included in other behavioral analyses. We scored all behaviors of both mothers and pups 30 min prior to and post birth with focal sampling [21]. In total, 142 hours of video were analyzed from seven nights. Due to technical issues, the focal roost was not always recorded for all three periods on the same day. We scored the arrival period on six, the daytime period on five, and the departure period on five of the dates between 6 June and 21 June 2014.

To calculate activity budgets, we extracted the frequency of each behavior in every 10-scan session from SolomonCoder. Then we averaged this proportion (x/10) for each individual for all three time periods and reported values as percent of scans. As individuals could not be followed for longer than one day-roosting period, we analyzed behavior at the group level. Social interactions were analyzed as a single unit of behavior. The number of sessions per arrival and departure period varied as the roosting group fluctuated their arrival and departure times by more than 15 min between dates.

All methods conformed to the ASAB/ABS Guidelines for the Use of Animals in Research and were approved by the Autoridad Nacional del Ambiente (now Ministerio del Ambiente, SE/A 58–12, SE/AP-4-13, SE/A 88–13, SE/AP 12–14, SE/A 73–14), and the Institutional Animal Care and Use Committee of the Smithsonian Tropical Research Institute (2012-0601-2015).

## Results

### Birthing season

**Fig 1:**
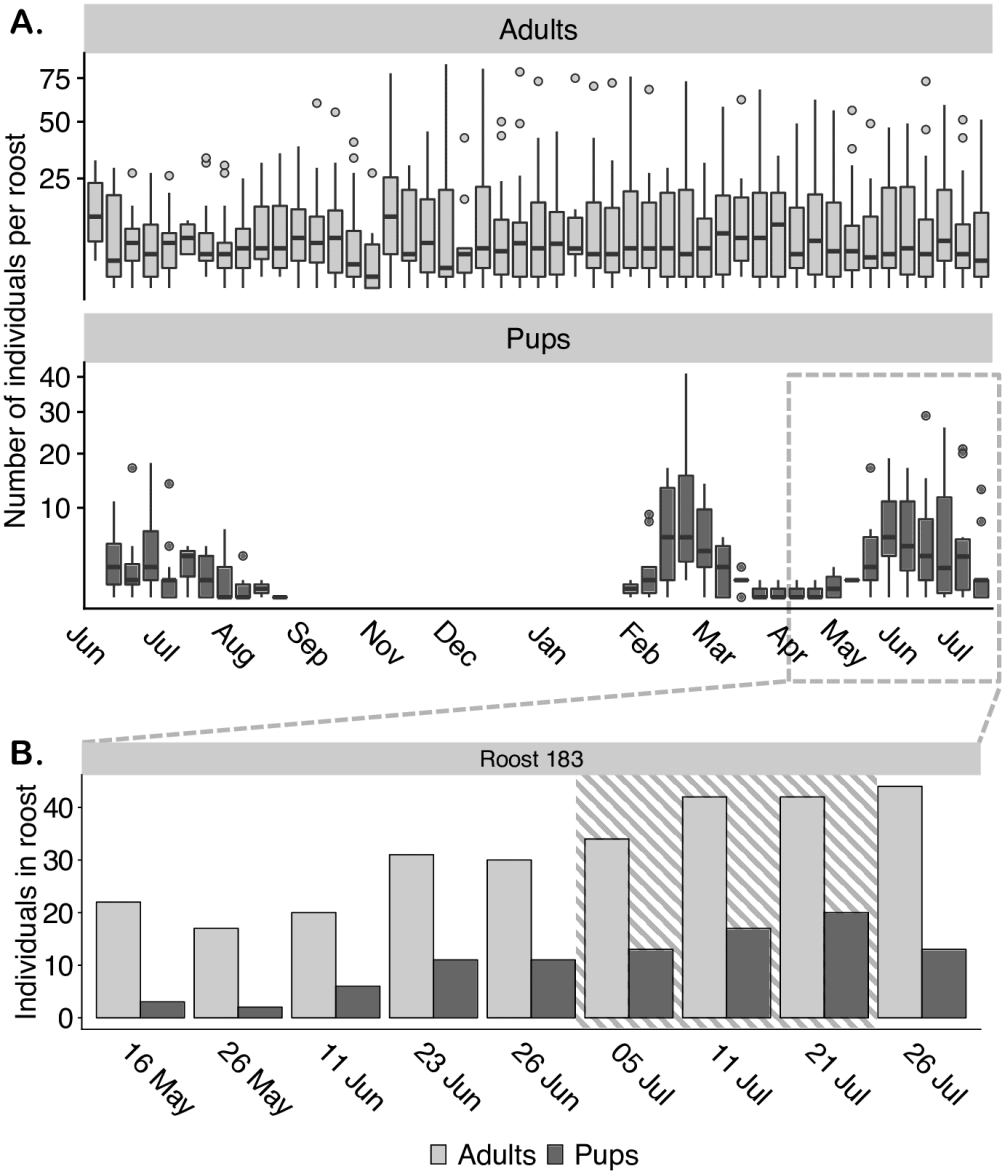
*Uroderma bilobatum* exhibits two birth peaks per birthing season. (A) Number of adults (light gray) and pups (dark gray) present per roost (house) in Gamboa from 10 June 2013 to 26 July 2014. Each box represents 30–67 houses. The horizontal line through the box represents the median. The lower and upper box edges correspond to the first and third quartiles. The lower and upper whiskers correspond to the smallest and greatest values, no more than 1.5 * interquartile range. The colored circles represent outliers. The y-axis was transformed to a square root scale. (B) Number of adults (light gray) and pups (dark gray) present at one roost (house) made up of multiple maternity groups from 15 May 2014 to 26 July 2014. Roost videos of a single maternity group (area of dashed lines) spanned a 2-week period during peak pup presence in this roost, from 6 July to 21 July 2014.

We surveyed 67 maternity roosts from 10 June 2013 to 26 July 2014 (Fig 1A) in an area of 0.25 km^2^ (S1 Dataset). There were two partial birthing seasons observed during this time, June through August 2013 with one peak in pup presence, and February through July 2014 with two peaks (Fig 1A). During the birthing season in which roost video recordings were made, June through July 2014, 20 roosts contained pups. Adult presence in these roosts ranged from two to 73 individuals, median of 15. Mothers were never seen with more than one pup. Pup presence in these maternity roosts ranged from one to 29 individuals, with a median of four. Pup presence peaked one month into this birthing season after which pups started becoming indistinguishable from adults in our survey. Our focal maternity roost was never without pups during the 2-peak birthing season from February through July but reached a minimum on 26 May 2014 (Fig 1B). Roost videos captured a 2-week period around peak pup presence in the focal roost from 06 July to 21 July 2014.

### Parturition

We recorded the last birth for this maternity group on day 29 of the birthing season. We observed three earlier births at another roost not included in our other behavioral analyses (S1 Video) (S2 Dataset). Mothers did not isolate themselves but stayed in the maternity roost to give birth. They spent 15.3 ± 15.3 (mean ± SD) of the 30 minutes prior to birth in contact with other groupmates, 15.5 ± 3.7 minutes resting and 3.9 ± 3.4 minutes grooming. They gave birth in the same position that they were roosting, hanging by their feet, except they frequently hung from one foot instead of two (one foot = 11.6 ± 9.8 minutes). Other pre-birth behavioral changes included sniffing and licking the genital region and holding their forearms out from the body instead of tightly against the body as they are typically held. Births lasted 1.63 ± 0.74 minutes from the time any part of the pup could be seen to full emergence. All pups were delivered head first. Mothers spent 10.0 ± 13.9 of the 30 minutes post-birth in contact with other roostmates and 6.1 ± 4.7 minutes resting. After birth they groomed their pups more than themselves (mother autogrooming = 9.3 ± 8.1 min; pup allogrooming 13.3 ± 4.8 min). One mother was observed delivering and consuming her placenta 3.9 hours after birth.

Roost mates did not show interest in birthing mothers or newborns except on one occasion. Two births occurred on the same day within 16.6 minutes of one another. The females were roosting adjacent to each other and the second female to give birth was highly interested in the first birth (S1 Video). She spent 12.6 of the 30 minutes prior to her own birth sniffing and licking the first emerging fetus and grabbing it with her forearm, pulling it closer to her mouth to lick. The birthing mother showed aggression towards this interference a few times, but mostly permitted the other mother’s actions.

### Morphological development in pups

**Fig 2:**
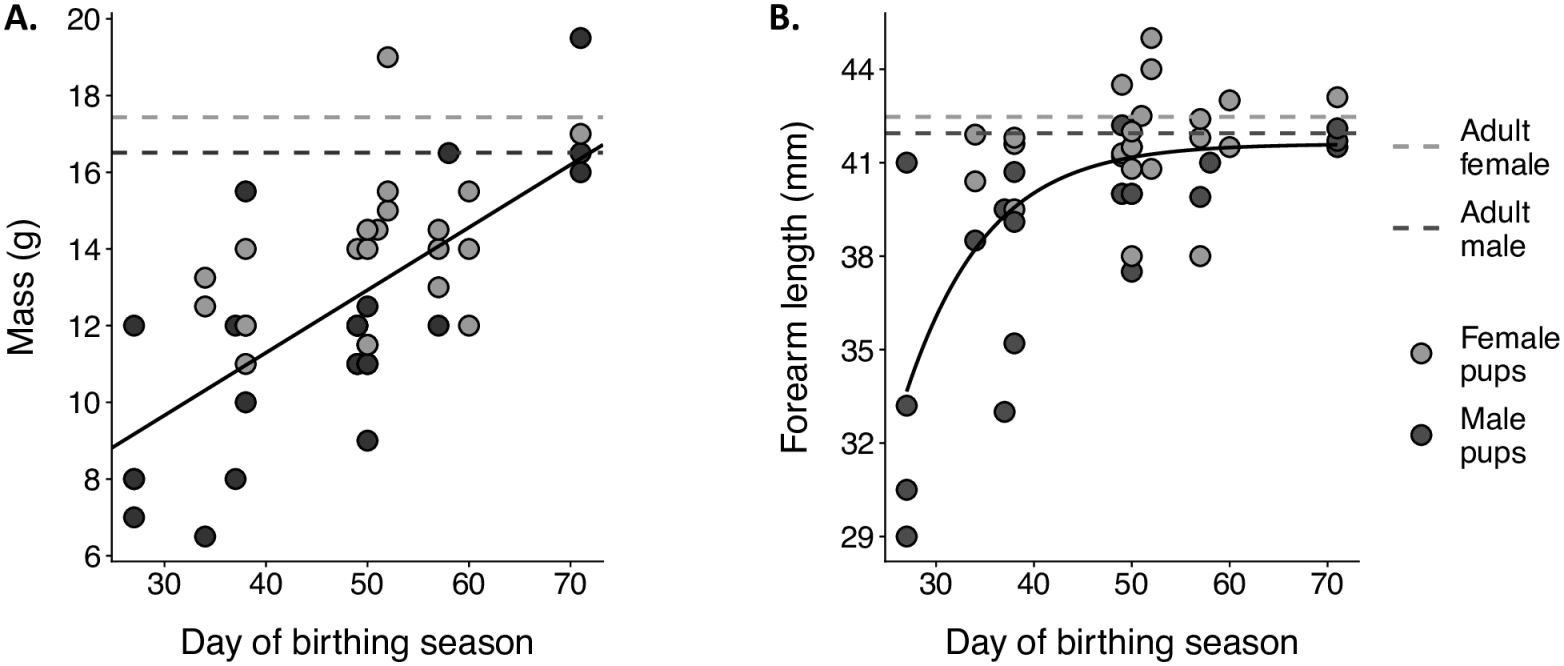
Pups reach adult forearm length more quickly than adult mass. Mass in grams (left) and forearm length in millimeters (right) of 41 *Uroderma bilobatum* pups captured throughout the birthing season. Females are shown in light gray circles and males in dark gray circles. The light gray line represents average adult female size and the dark gray line, male size for this population in Gamboa.

We measured both mass and forearm length of 44 pups between the estimated ages of 27 to 71 days (estimated from roost survey data) (S3 Dataset). On average, pups reached mean adult mass (females: 17.3 ± 1.4 g, males: 16.5 ± 1.1 g) at 77.59 days for females and 71.93 days for males (Fig 2A). A Gompertz growth model shows that pups reached mean adult forearm length (females: 42.4 ± 1.3 mm, males: 41.9 ± 1.1 mm) at 34.50 ± 7.59 days (SE) days with an initial growth rate of 2.06 ± 0.76 (SE) mm day^-1^ (Fig 2B).

### Maternal support and pup independence

Group size for the video-recorded maternity group ranged from 11 to 13 adults and 11 to 13 pups. Mothers were never seen with more than one pup. Adults that did not have a pup were only observed with the group on a few occasions and never stayed for the entire day. Pups varied in age by up to 27 days as the first pups were seen in the focal roost on 11 June 2014 and the last pup in the video recorded maternity group was born on 08 July 2014.

Pups typically roosted tightly with their mother while clinging to her body or hanging by their own feet. Pups were never present in the roost alone overnight but were either carried away from the roost by their mother or later departed on their own. We never observed mothers provision their pups with solid food in the roost. On day 27 of the birthing season we observed two mothers return to the roost between 3.1 and 3.2 hours after the start of evening emergence to nurse pups. We only observed aggression between mothers and their own pups on two occasions when pups tried to suckle and mothers responded by nipping. On some occasions we observed mothers aggress towards pups that were not their own. This occurred when pups tried to shift in the roost and move around the female or when pups initiated an interaction (e.g., tried to suckle). These aggressive interactions usually occurred when the pup’s own mother was absent. This aggression occurred most frequently in the arrival period when pups arrived before their mothers and were without them in the roost or trying to reunite with their mothers in the roost. We did not observe allogrooming or allonursing between mothers and pups that were not their own.

The percent of time that mothers were present during the departure period was lower and more variable than during the arrival and daytime periods (mean ± SD; arrival = 95.8 ± 19.9, daytime = 96.8 ± 17.4, departure = 45.8 ± 49.3) (S4 Dataset). Thus, we report activity during departure separately in the next section. During the arrival and daytime periods, pups were most frequently suckling from their mothers, but decreased the percent of time suckling from day 25 to day 40 (mean ± SD percent of scans; day 25: arrival = 92.7 ± 22.4, daytime: 94.0 ± 19.3; day 40: arrival = 89.5 ± 29.8, daytime: 76.3 ± 41.4). We observed mothers moving away from their pups by flying or shifting to the other side of the roost or flying away from the roost altogether. Mothers decreased the percent of time spent in the roost (and thus availability for pup suckling) from day 25 to day 40 (day 25: arrival = 96.6 ± 18.3, daytime: 98.7 ± 11.0; day 40: arrival = 90.3 ± 29.8, daytime: 91.6 ± 27.7). On day 40 one pup never suckled from its mother. We could not determine if its mother was present in the roost at all on that day.

**Fig 3:**
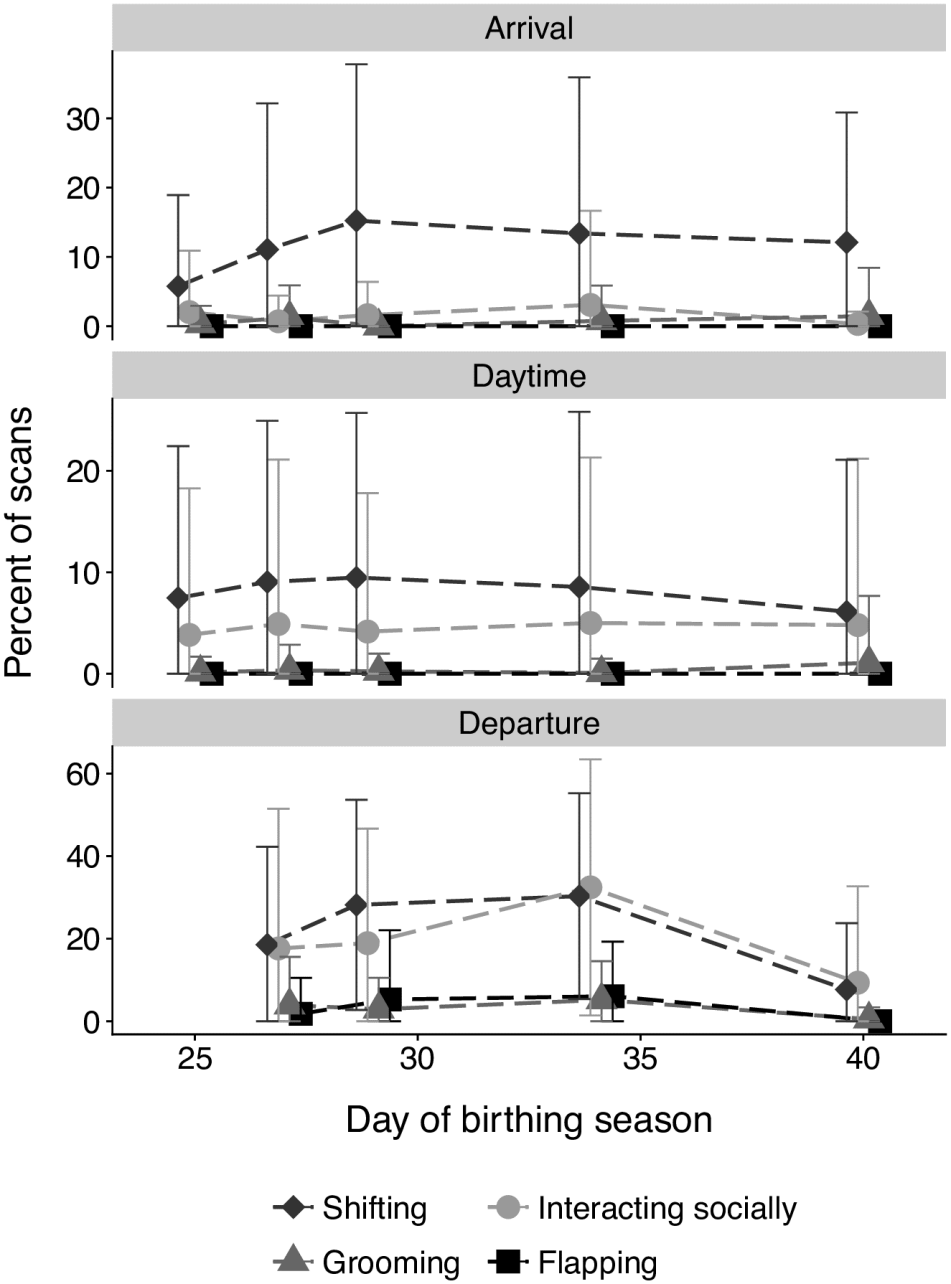
Pups experience greatest behavioral development in the departure period coinciding with fledging. Shifting behavior (diamonds), grooming behavior (circles), social interactions (triangles), and flapping behavior (squares) calculated as mean percentage of scans with observed behavior per coding session for each pup, averaged for pups as a group per day of the birthing season for each time period. Error bars represent ± standard deviation.

Mothers groomed their pups more than pups groomed themselves during the arrival period but the opposite was observed during the daytime period. Mothers decreased the percent of time spent in pup allogrooming and pups increased autogrooming during the day period from day 25 to day 40 (maternal allogrooming day 25: arrival = 3.6 ± 12.1, daytime: 2.2 ± 10.5; day 40: arrival = 2.4 ± 11.0, daytime: 1.2 ± 7.2; pup autogrooming day 25: arrival = 2.0 ± 8.9, daytime: 3.8 ± 14.4; day 40: arrival = 0.3 ± 1.8, daytime: 4.8 ± 16.4) (Fig 3).

Pup activity (grooming plus shifting) was nearly equal during arrival and daytime periods but pups were more frequently shifting than grooming. Shifting behavior increased while grooming decreased during the arrival period from day 25 to 40 (day 25: arrival = 5.8 ± 13.2, daytime: 7.5 ± 15.0; day 40: arrival = 12.1 ± 18.7, daytime: 6.1 ± 15.0) (Fig 3). Although infrequent, social interactions increased from day 25 to day 40 (day 25: arrival = 0.3 ± 2.6, daytime: 0.1 ± 1.5; day 40: arrival = 1.5 ± 7.0, daytime: 1.1 ± 6.6) (Fig 3).

**Fig 4:**
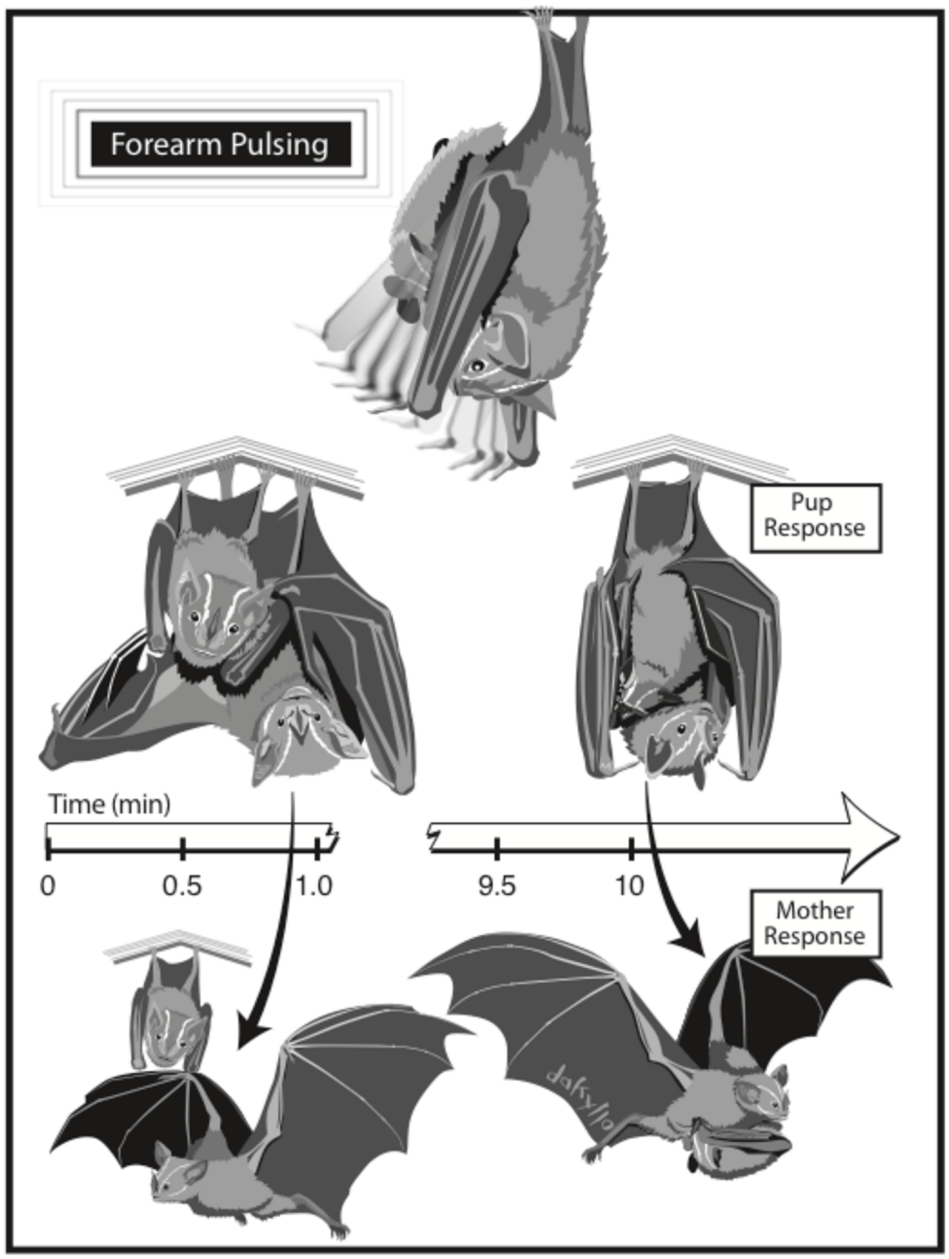
Mothers exercise stereotyped tactile stimulation on pups through forearm pulses. When mothers pulsed forearms on pups, pups responded in one of two ways: 1) detaching from teats, or 2) continuing suckling. Subsequently, mothers responded in one of two ways: 1) departing from roost leaving pup behind, or 2) departing from roost carrying pup. Mothers that departed from the roost without their pups spent < 1 minute intermittently pulsing forearms while mothers that carried pups away from the roost spent > 10 minutes intermittently pulsing forearms. Illustration by Damond Kyllo.

Mothers interacted with pups during the departure period through tactile stimulation (Fig 4). Before departing, the majority of mothers pulsed forearms on pups (31 of 41 departures preceded by forearm pulses) (S5 Dataset). The majority of pups responded to forearm pulses by detaching from their mothers’ teats and hanging by their own feet if not already (for 23 of 31 events of forearm pulsing, pups responded by detaching from mother). Pups that remained attached despite pulses were carried away from the roost by their mother.

When pups did not respond to pulses by detaching from mothers’ teats, mothers interacted with pups for longer periods of time (no detachment ≥10 min; detachment ≤1 min) (S2 Video) and spent a greater percentage of that time pulsing forearms relative to other activities like grooming themselves, their pups, or stretching (no detachment = 76% pulsing; detachment = 34% pulsing). Some pups detached and reattached several times during the interaction period, but overall pups reduced the frequency of re-attaching to mothers’ teats as they aged (mean number of reattachments on day 25 = 1.1; day 34 = 0.5). Forearm pulses were observed outside of the departure period on three occasions, but all in the same context of pups detaching from mothers.

When emerging from the roost at dusk, mothers not carrying pups left first, then mothers carrying pups, and lastly pups departing independently (S6 Dataset). Maximum pup latency to depart in a 1-week recording period decreased from 7.49 hours on day 27 to 1.40 hours on day 34. Mother-pup pair latency to depart increased variance, from a range of 3.17 min on day 27 to 20.41 min on day 34. Mothers leaving alone did not differ between days in latency to depart during this time period. Day 40 was not included because all roosting individuals appeared to have been startled out of their roost in the span of a few seconds, making their order of departure not comparable to other days.

From day 27–34, pups were more frequently active during the departure period (arrival = 13.1 ± 22.6; daytime = 13.0 ± 23.9; departure = 45.9 ± 39.1) (Fig 3). They increased activity for shifting, grooming, social interaction, and flapping behaviors over these days (shifting = 18.6 ± 23.7 to 30.3 ± 24.9; grooming = 17.6 ± 33.8 to 32.4 ± 31.0; social = 3.9 ± 11.7 to 5.2 ± 9.4; flapping = 1.8 ± 8.7 to 6.1 ± 13.2) (Fig 3). On day 40, activity for all behaviors decreased (shifting = 7.7 ± 16.1; grooming = 9.3 ± 23.4; social = 0.5 ± 2.9; flapping = 0.0 ± 0.0) and total activity became more similar to the arrival and daytime periods (arrival = 12.4 ± 19.6; daytime = 10.9 ± 25.0; departure = 17.0 ± 30.9) (Fig 3).

The majority of pups already flew away from the roost on their own at evening emergence on day 27, but the percentage increased slightly by day 35 (day 27 = 73% of pups; day 35 = 83%) (S7 Dataset). Most pups were carried into the roost by their mothers at dawn arrival on day 25 and the percentage decreased by day 40 (day 25 = 92%; day 40 = 42%), meaning more pups were flying back into the roost on their own. Sometimes pups and mothers arrived at the same time, flying into the frame just seconds apart. Other times pups arrived before mothers in the roost.

## Discussion

In accordance with other bat species studied to date, *Uroderma bilobatum* undergoes rapid early development, which is comparable to similar-sized mammals but exceptional compared to mammals of similar lifespan. From birth to fledging, mothers and pups exhibit behavioral adaptations that support their aerial lifestyle. Between weeks four and five of the birthing season, pups show behavioral development by suckling less and beginning to fly from and to the roost independently. Mothers exercise stereotyped tactile stimulation on pups through forearm pulses and leave pups behind in the roost, which may initiate fledging and weaning. Mothers also assist the fledging process by carrying pups back up into the roost when they have flown from the roost on their own.

### Birthing season

In our study site, *Uroderma bilobatum* exhibits seasonal bimodal polyoestry as previously reported for other Central American populations [16,22] with birth peaks in March and July. Each year, females produce two pups consecutively followed by a period of reproductive inactivity. Parturition coincides with seasons of highest fruit production, in the middle of the dry season and at the start of the rainy season [22,23]. Increased food abundance can benefit both lactating mothers and developing pups by reducing travel distance to food sources. Mothers can allocate energy from flying to producing milk, and pups can more easily reach foraging areas while honing flight skills [24].

### Parturition

Bats face the unique challenge of giving birth while hanging. We observed the same birthing posture for all four *U*. *bilobatum* as had been previously described for *Artibeus jamaicensis* and another unidentified phyllostomid [25,26]. Maintaining the same position for giving birth as for resting, with a slight modification of hanging by one foot instead of two, may be a typical birthing posture assumed by phyllostomids. In contrast, many vespertilionids reverse their roosting position to give birth in a head-up, feet-down posture, and some records describe their use of their uropatagium to form a basket that prevents the pup from falling when born [27,28]. *Uroderma bilobatum* deliver their pups head first like pteropodids and rhinolophids, while most vespertilionids and molossids deliver pups in a breech position [27]. The only two records for phyllostomid births report one of each. Our observations suggest that headfirst delivery is a more common birth position for Phyllostomidae.

The pups were very active during the last stages of birth. Once their shoulders and forearms were free, they participated in final delivery with frantic movements and lateral wing movements. Jones [26] reported similar precocial behavior for newborn *A*. *jamaicensis*. All birthing mothers licked their pups as they emerged which may break and remove the amniotic sac [27]. Pups also actively moved in search of teats and started suckling within seconds after birth, which seems to be common for all bats [27]. Finally, like *A*. *jamaicensis*, *U*. *bilobatum* pups were born with eyes open and fur already covering the body [26].

Roost mates generally did not react to bats giving birth and mothers spent less time in contact with roost mates after birth than before birth. Lewis [19] did not observe any births directly, but noted a lack of interest by roost mates towards newborn pups in wild *U*. *bilobatum* maternity groups. In our study, we only observed an interested female on one occasion which spent a considerable amount of time licking another’s pup during parturition and after birth. Kunz et al. [29] observed a similar incident of allogrooming during parturition in captive *Pteropus rodricensis*. The female we observed interacting with the birthing mother gave birth herself less than 20 min later suggesting she may have been preparing for her own birth in some way, rather than assisting her group mate. We observed placentophagia for one birth almost four hours after delivery of the fetus. Although this is longer than typical mammalian patterns, both previous records of phyllostomid births report delivery of the placenta between four and 9.5 hours after fetus delivery and between 30 min and six hours for other bat species [25–27].

### Morphological development in pups

Overall, postnatal growth of *U*. *bilobatum* is similar to the broad pattern observed for Chiroptera. Pups grow rapidly in the first weeks of life, reaching nearly adult size while still receiving maternal support [10]. Forearm length increases faster than mass, facilitating the ontogeny of flight [30]. On average, pups in our study appear to reach mean adult forearm length at 34 ± 7 days into the birthing season, which places *U*. *bilobatum*, as a medium-sized phyllostomid, between values reported for a smaller and a larger related species. *Dermanura watsoni* (approx. 74% smaller than *U*. *bilobatum*) reaches a growth asymptote around 30 days and *Phyllostomus hastatus* (approx. 48% larger than *U*. *bilobatum*), around 40 days [24,31]. Our conservative age estimation based on pups’ appearance in roosts within a 2-week time span may have overestimated age of individuals, so we do not report these data as representative for the species. Forearm growth was curvilinear while mass increased linearly without an asymptote, similar to *Dermanura watsoni*, which continue to rapidly accumulate mass after reaching adult forearm length [24]. *Uroderma bilobatum* pups seem to confirm the pattern that tropical species grow more slowly than temperate species, which are more constrained by seasonality [10].

### Maternal support and pup independence

Maternal support declined with progression of the birthing season. Between days 25 and 40, mothers groomed their pups less, and spent less time in direct contact with them, reducing potential thermoregulatory assistance and availability for nursing [6]. By day 40, one pup did not receive any maternal support. Maternal support also declines for *Artibeus watsoni*, another phyllostomid, after 25 days of age [24]. *Plecotus auritus* and *Myotis formosus*, both vespertilionids, experience more rapid reductions in maternal care, with mothers spending less than 25 percent of time in contact with pups by 40 days of age, compared to 75 percent for *U*. *bilobatum*, although in our study not all pups were yet that age [14,32]. In accordance with slower morphological growth, *U*. *bilobatum* may have slower behavioral development than most temperate species.

Mothers carried pups away from the roost until they were capable of emerging on their own. We assume *U*. *bilobatum* transports non-volant pups to secondary roosting locations at night. Lewis [19] also observed *U*. *bilobatum* carry pups away from the day roost and suggested mothers were transporting young to night roosts because they caught females carrying pups early but not later in the evening. Pup transport during the foraging period has been reported for many frugivorous and nectarivorous bats, but is rare in insectivorous bats [6,12]. Most reports cannot confirm whether young were being carried to alternative roosts or to foraging grounds. Pup transport is expected to greatly increase costs of flight for the mother in terms of wing loading, maneuverability, foraging efficiency, and energetics [7,33,34]. In our study site, despite being the second-most common bat caught at fruiting fig trees [35], *U*. *bilobatum* were never caught in foraging areas carrying pups. Moving young to secondary roosts may reduce their exposure to predators or prevent predators from learning the locations of defenseless pups, while minimizing flight costs to mothers [19,36,37]. On day 28, two mothers returned to the day roost to provision volant pups with milk three hours after dusk emergence. If mothers transport non-volant pups to secondary roosts while they forage, they may be returning to these locations to provision pups before they are weaned. This behavior has been reported for *Myotis lucifugus* and suggested, but not confirmed, for phyllostomids [38].

Pup independence increased with progression of the birthing season. By day 40, they spent less time suckling, and one individual did not suckle or roost with its mother for the entire day. We did not observe mother-pup aggression to encourage early stages of pup independence and weaning. Mothers did, however, intermittently separate themselves from their pups to other areas of the roost during the day. Aggressive mother-offspring conflict during weaning seems to be rare in most bat species compared to other mammalian orders [6,39]. Perhaps bats can initiate weaning without aggression due to the limited mobility of their non-volant young. Our observation period did not capture weaning for the whole maternity group. The weaning process usually extends beyond fledging and the start of pups foraging on their own [6]. It seems that by day 40 of the birthing season this process has begun for some pups while others are developmentally further behind, likely due to age differences.

Non-aggressive mother-offspring conflict may also take place as mothers increasingly left pups in the roost during the departure period rather than carrying them away. Here we observed a novel behavior of mothers interacting with pups through the use of forearm pulses. Pulses did not appear to disturb pups, yet most pups responded by detaching from teats, allowing mothers to fly away from the roost without them. This may facilitate an important stage of the developmental process, where mothers leave pups behind in the roost, forcing them to emerge on their own rather than being transported to a secondary roosting location. Tactile stimulation is an important sensory modality between mothers and offspring for other mammalian species [40], but has not been directly studied in bats [5]. Reports of tactile communication in bats are limited to its role in mating displays for some species [41]. We postulate this forearm pulsing behavior as a form of tactile communication between mothers and pups, stimulating the important developmental transitions of fledging and weaning with reduced conflict.

As pups were left behind in the roost, they delayed their own emergence. Some studies have considered emergence of juveniles as they develop, but none have reported such drastic changes as we observed in *U*. *bilobatum*. Duvergé et al. [42] reported a maximum of 2.1 hours difference between mother and juvenile *Eptesicus fuscus* while we report a maximum of 7.49 hours. Kunz [43] reported juveniles adjusting departure time to that of mothers, but from a maximum of less than one hour for *Myotis velifer*. Delayed emergence has been explained in the context of decreased predation risk for juveniles who are not yet coordinated flyers [1,44]. We only found one report of a frugivorous bat where juveniles emerged later than females during the birthing season, but the study gave no detail of emergence time development [45]. Frugivores are not constrained by short time windows of prey availability, which may allow juveniles to afford later emergence times while developing. However, for animals that depend on widely distributed and unpredictable fruit resources, further energetic constraints [46] may limit developmental rates [47–49]. Alternatively, pups may not be foraging at this stage, but rather making practice flights while still receiving nutritional support from mothers.

On nights where pups were left in the roost by their mothers and remained for some time before emerging on their own, we observed increased activity in grooming, shifting, social interaction, and flapping behaviors. Other mammal infants exhibit similar behavior when separated from their mothers. Rat pups show increased exploratory behavior, sniffing, and self-grooming when isolated [50]. Hyperactivity is also a behavior associated with the “protest” stage of mother-infant separation in human and non-human primate infants [51,52]. Bat pups may be responding with similar behaviors when left alone in the roost. By day 40, activity levels were reduced and comparable to activity levels during the arrival and daytime periods. The reduction of these behaviors as pups aged suggests this is an element of behavioral development in the transition to emerging from the roost and foraging on their own.

Wing flapping behavior was also an important component of increased activity during the departure period. Wing flapping was observed for several pups during one week (day 27 to day 34) and ceased by the end of the following week. Pups rapidly flapped wings, even generating lift, but stayed in the roost by clinging to the roof with their feet. We were not able to observe directly if this behavior progressed into practice flights later in the night because of the limits of the video frame, but this seems likely considering our observations of a two-stage fledging process (discussed below). Wing flapping behavior has been described for many species during flight development, reviewed in [13]. Hughes et al. [13] suggested stretching and flapping may be just as prevalent as practice flights for pre-flight training and Powers and Kunz [53] argued that these movements likely play important roles in muscle, bone, and joint development. Captive juvenile *Noctilio albiventris* began wing flapping between 31 and 34 days old, similar to *U*. *bilobatum* in our study [54].

After pups emerged from the roost on their own, we observed some being carried back into the roost by their mothers on subsequent mornings. This suggests that fledging occurred in a two-stage process, where pups were at first only capable of flying away from the roost, but perhaps not sufficiently coordinated to maneuver back up into the roost. We cannot say where and how far pups flew in this practice stage, or how mothers relocated pups before bringing them back into the roost. At the second stage, pups were capable of flying back up into the roost on their own. By day 40, half of the pups flew back into the roost, suggesting these individuals were fully fledged. Forty days into the birthing season is comparable to age at fledging for *Noctilio albiventris* (35–44 days old) [54]. Most pups were still suckling on this date, suggesting that pups do not forage during first flights, but hone flight skills while still receiving nutritional support from mothers.

In sum, this study details the behavioral and morphological changes accompanying the rapid development of *U*. *bilobatum* pups. This work highlights the importance of observational studies to identify novel behaviors and strategies employed by bats to facilitate life on the wing.

## Supporting information

S1 Dataset Survey of mothers and pups roosting under houses in Gamboa. (CSV)

S2 Dataset Focal sampling of parturition behavior. (CSV)

S3 Dataset Mass and forearm length of pups caught in Gamboa. (CSV)

S4 Dataset Instantaneous scan sampling of mother and pup behavior in day roost. (CSV)

S5 Dataset Focal sampling of forearm pulsing behavior. (CSV)

S6 Dataset Latency to depart from roost for each mother, mother-pup pair, or pup. (CSV)

S7 Dataset Frequency of pups being carried by mother or flying on their own for arrival to and departure from roost. (CSV)

S1 Video Birth of *Uroderma bilobatum* in the wild. (MP4)

S2 Video Maternal tactile stimulation of pups through forearm pulses. (MP4)
